# Chemical Reaction and Internal Heating Effects on the Double Diffusive Convection in Porous Membrane Enclosures Soaked with Maxwell Fluid

**DOI:** 10.3390/membranes12030338

**Published:** 2022-03-18

**Authors:** Dhananjay Yadav, Maimouna Al-Siyabi, Mukesh Kumar Awasthi, Salma Al-Nadhairi, Amna Al-Rahbi, Maryam Al-Subhi, Ravi Ragoju, Krishnendu Bhattacharyya

**Affiliations:** 1 Department of Mathematical & Physical Sciences, University of Nizwa, Nizwa 616, Oman; 09946591@uofn.edu.om (M.A.-S.); 14225152@uofn.edu.om (S.A.-N.); 19482031@uofn.edu.om (A.A.-R.); 09692056@uofn.edu.om (M.A.-S.); 2 Department of Mathematics, Babasaheb Bhimrao Ambedkar University, Lucknow 226025, India; mukeshiitr.kumar@gmail.com; 3 Department of Applied Sciences, National Institute of Technology Goa, Goa 403401, India; ravi@nitgoa.ac.in; 4 Department of Mathematics, Institute of Science, Banaras Hindu University, Varanasi 221005, India; krishmath@bhu.ac.in

**Keywords:** convective instability, Maxwell fluid, porous membrane enclosure, mass transfer, internal heating, chemical reaction

## Abstract

In this paper, the joint impact of the interior heating and chemical reaction on the double diffusive convective flow in porous membrane enclosures soaked by a non-Newtonian Maxwell fluid is investigated applying linear and nonlinear stability techniques. The porous enclosures are square, slender and rectangular. Using the linear stability analysis, the expression for the critical thermal Rayleigh–Darcy number, above which the convective movement occurs, is derived analytically in terms of associated physical parameters. A nonlinear stability examination reliant on the Fourier double series is executed to calculate the convective heat and mass transports of the arrangement. It is observed that the pattern of convective activity is oscillatory only in the occurrence of a relaxation parameter and the threshold value of the relaxation parameter for the occurrence of the oscillatory pattern depends on the other physical parameters. The onset of convective instability accelerates with the increasing chemical reacting parameter, the interior heating parameter, the solute Rayleigh–Darcy number, the Lewis number, the Vadasz number, and the relaxation parameter, while it delays with the heat capacity ratio. The convective heat and mass transfers increase with the solute Rayleigh–Darcy number, the Vadasz number, the relaxation parameter, and the aspect ratio (for rectangular enclosure), while it decreases with the heat capacity ratio and the aspect ratio (for slender enclosure). Additionally, the convective heat transfer enhances with the interior heating parameter, while the convective mass transfer enhances with the chemical reacting parameter and the Lewis number. The effects of Vadasz number, heat capacity ratio, and relaxation parameter are witnessed only on the oscillatory pattern of convection and unsteady convective heat and mass transfers. Further, some existing literature results are compared with the current findings.

## 1. Introduction

Double diffusive convective motion is encouraged by two components whose densities are different. These density gradients result in two different diffusion rates which are very crucial for this type of convection. Convection that occurs due to concentration and temperature gradients has gained popularity in previous years because of its realistic applications in science and engineering. Some of the vital sectors of relevances in engineering contain foodstuff and chemical processes [1,2,3,4], casting of metals [5,6,7], crystal growth [8], petroleum production [9,10,11], biomechanical and geosciences applications [12,13,14,15]. The problem of the double diffusive convective progress in a porous layer was explored by Poulikakos [16]. He derived the boundaries describing the areas of direct and overstable styles in terms of critical parameters. The anisotropic effect on the double diffusive convective movement in a porous surrounding substance was explored by Gaikwad et al. [17]. They considered the impacts of anisotropy parameters, solute Rayleigh number, the Soret, and the Dufour factors on the stationary and oscillatory convections as well as on the warmth and mass carrying. Kuznetsov and Nield [18] investigated the heterogeneity effect on the beginning of double diffusive convective movement analytically exploiting linear stability theory. The impact of throughflow on the onset of double-diffusive motion in a permeable layer was explored by Kiran [19] and Shivakumara and Khalili [20]. They found that the consequence of throughflow has either to become stable or to unsettle the arrangement. It depends on the direction of throughflow. Javaheri et al. [21] deliberated the double diffusive convective flow as a purpose for the geological congregation of carbon dioxide. The exponential discrepancy of boundary conditions on the unsteady double diffusive natural convective progress inside the porous enclosures was investigated by Al-Mudhaf [22]. Altawallbeh et al. [23] calculated the power of internal heating on binary instability in a permeable layer. They obtained the critical Rayleigh number and wave number for the stationary and oscillatory mode of convections exploiting the linear hypothesis. Malashetty and Biradar [24] examined the consequence of chemical feedback on the binary diffusive instability in a porous medium layer. They found that the chemical feedback may have a stabilizing or destabilizing influence on the stability criterion. An outstanding review of the studies associated with the double-diffusive instability has been provided by Nield and Bejan [13], Yadav [25], Mojtabi and Charrier-Mojtabi [26], and Vafai [27].

It is known that fluids that take place in the majority of the above-mentioned applications and in nature exhibit non-Newtonian fluid features [28,29,30,31,32,33,34,35,36,37,38]. The problem of double-diffusive instability in a porous medium layer drenched by non-Newtonian fluid taking an Oldroyd model was explored by Malashetty and Swamy [39]. They derived the arrival conditions for stationary, oscillatory, and finite amplitude convective motions analytically. They found a contrast amid the progression of thermal transmission, solute transmission, and viscoelasticity to establish the convective motion as an oscillatory pattern. Malashetty et al. [40] and Kumar and Bhadauria [41] extended this problem with the thermal non-equilibrium effect. The linear stability examination of a Maxwell liquid with double-diffusive motion was undertaken by Awad et al. [42]. They demonstrate that the critical Darcy–Rayleigh number reduces with the relaxation time. Wang and Tan [43] inspected the dual-diffusive instability for non-Newtonian liquid in an absorbent medium considering an amalgamation of Maxwell and Darcy models. They observed that the Soret outcome and relaxation time sped up the onset of convection. The consequence of interior heating on the beginning of double diffusive instability in a coupled stress non-Newtonian liquid flooded porous layer was scrutinized by Gaikwad and Kouser [44]. They found that the arrival of both stationary and oscillatory convective movement is increased by the interior Rayleigh number. Gaikwad and Dhanraj [45] scrutinized the combined weight of anisotropic and interior heating on the binary flow in a non-Newtonian Maxwell liquid flooded permeable layer. They observed that the inner Rayleigh number, mechanical anisotropy factor, and relaxation parameter sped up the start of convective activity, while the thermal anisotropy factor delayed it. Very recently, the impact of larger frequency pulsation in the gravity force on the double-diffusive convective activity with non-Newtonian viscoelastic liquid-filled porous matrix was inspected by Zhao et al. [46].

From the literature inspection, clearly no effort has been made to determine the mutual impact of chemical response and interior heating on the beginning of double diffusive convective motion in a permeable layer flooded by a non-Newtonian fluid. However, there are several practical applications in enhanced oil recovery systems (such as during polymer-flooding practices), a packed bed reactor, chemical privilege equipment, food manufacturing, metal casting procedures, and geophysical arrangements where the porous material may offer its source of warmth and the chemical reaction can take place among the chemical species in the porous substance and the non-Newtonian fluid. Therefore, the present effort intends to inspect the mutual effect of the interior heating and chemical reaction on the double diffusive convective motion in permeable enclosures flooded by a non-Newtonian fluid applying linear and nonlinear stability techniques. To model the non-Newtonian behavior of the fluid, the Maxwell model is used. This is a rate variety of non-Newtonian fluid models in which stress relaxation is known. The Maxwell fluid model acceptably describes the flow behavior of non-Newtonian fluids consisting of a substructure, for instance lubricants with polymer additions, electro-rheological fluids, liquid crystals, blood, and suspension fluids [47,48,49,50]. This work is presented as follows: In Section 1, an introduction of the problem under investigation is provided. In Section 2, the mathematical formulation of the problem is presented. The perturbation equations are obtained in Section 3. In Section 4, the conditions for the start of binary convective flow are derived. The convective heat and mass transports are derived in Section 5. Section 6 presents key results and discussion. At last, this work is completed with a conclusion in Section 7.

## 2. Mathematical Formulation

**Figure 1:**
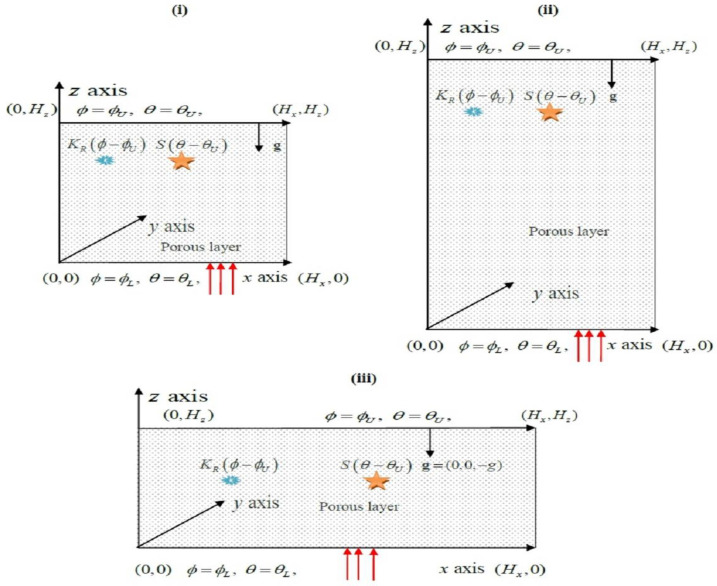
Physical configuration of the problem: (**i**) square enclosure, (**ii**) slender vertical enclosure, and (**iii**) rectangular.

The system examined is a non-Newtonian Maxwell fluid-saturated porous cavity of length $H_{x}$ and width $H_{z}$ with a third dimension infinitely extended so that the fluid flow and heat and mass transport can be taken as two dimensional [29,51], as demonstrated in Figure 1.

It is assumed that the porous cavity is subjected to an internal heat supply of strength S and first-order chemical response of rate $K_{R}$ among a chemical genus in the permeable matrix and the Maxwell fluid. The temperatures $\theta_{L}$ and $\theta_{U}$, and solute concentrations $\phi_{L}$ and $\phi_{U}$, are consistently forced on the bottom and top boundary walls such that $\theta_{L}>\theta_{U}$ and $\phi_{L}>\phi_{U}$, while other boundary walls are accepted to be insulated and impermeable to mass transmission. It is specified that the porous cavity is homogeneous and in local thermal balance with Maxwell fluid.

The continuity equation for the Maxwell fluid in a porous cavity is [52,53,54]:

(1) $$\nabla .V_{D}=0.$$

Here, $V_{D}$ is Darcy’s velocity of the Maxwell fluid, $\nabla =i_{x}\frac{\partial }{\partial x}+i_{z}\frac{\partial }{\partial z}$ and, $i_{x}$ and $i_{z}$ are unit vectors in x and z paths.

The momentum equation by taking the Darcy rule for the porous medium, the Maxwell model for non-Newtonian fluid, and the Boussinesq approximation for density disparity with temperature and solute concentration in the buoyancy force is [55,56,57]:

(2) $$\frac{\mu }{K}V_{D}=\left(1+\gamma_{1}\frac{\partial }{\partial \tau }\right)\left[-\frac{\rho_{0}}{\varepsilon }\frac{\partial V_{D}}{\partial \tau }-\nabla P+\rho_{0}\left\{1-\beta_{\theta }\left(\theta -\theta_{U}\right)-\beta_{\phi }\left(\phi -\phi_{U}\right)\right\}g\right].$$

where, τ represents the time, θ symbolizes the temperature, ϕ represents the solute concentration, $\rho_{0}$ symbolizes the density at $\theta_{U}$, K represents the permeability of the porous medium, $\gamma_{1}$ symbolizes the stress relaxation feature time constant, μ symbolizes the viscosity of Maxwell fluid, P symbolizes the pressure, $\beta_{\theta }$ and $\beta_{\phi }$ symbolize the thermal and solute expansion coefficients, respectively.

The energy equation for a Maxwell fluid in a heat-generating permeable cavity is [31,58,59]:

(3) $$\left[{\left(\rho c\right)}_{E}\frac{\partial }{\partial \tau }+\left(\rho c\right)\left(V_{D}.\nabla \right)\right]\theta =k_{E}\nabla^{2}\theta +S\left(\theta -\theta_{U}\right).$$

Here, S symbolizes the power of the interior heating, $k_{E}$ symbolizes the effectual thermal conductivity of the porous medium, (ρc) and ${\left(\rho c\right)}_{E}$ symbolize the heat capacities of the Maxwell fluid and effectual permeable medium, correspondingly.

The conservation equation for the solute with the chemical reaction of rate $K_{R}$ in the porous matrix is [60]

(4) $$\left[\frac{\partial }{\partial \tau }+\frac{1}{\varepsilon }\left(V_{D}.\nabla \right)\right]\phi =D_{S}\nabla^{2}\phi +K_{R}\left(\phi -\phi_{U}\right).$$

Here, $D_{S}$ is the solutal diffusivity and ε is the porosity of the permeable medium.

On eliminating the pressure term and using the stream function χ as u=−∂χ/∂z and w=∂χ/∂x, the Equations (1)–(4) can be written as:

(5) $$\frac{\mu }{K}\nabla^{2}\chi =\left(1+\gamma_{1}\frac{\partial }{\partial \tau }\right)\left[-\frac{\rho_{0}}{\varepsilon }\frac{\partial }{\partial \tau }\left(\nabla^{2}\chi \right)+\rho_{0}\beta_{\theta }g\frac{\partial }{\partial x}\left(\theta -\theta_{U}\right)+\rho_{0}\beta_{\phi }g\frac{\partial }{\partial x}\left(\phi -\phi_{U}\right)\right],$$

(6) $${\left(\rho c\right)}_{E}\frac{\partial \theta }{\partial \tau }+\left(\rho c\right)\left(\frac{\partial \chi }{\partial x}\frac{\partial \theta }{\partial z}-\frac{\partial \chi }{\partial z}\frac{\partial \theta }{\partial x}\right)=k_{E}\nabla^{2}\theta +S\left(\theta -\theta_{U}\right),$$

(7) $$\frac{\partial \phi }{\partial \tau }+\frac{1}{\varepsilon }\left(\frac{\partial \chi }{\partial x}\frac{\partial \phi }{\partial z}-\frac{\partial \chi }{\partial z}\frac{\partial \phi }{\partial x}\right)=D_{S}\nabla^{2}\phi +K_{R}\left(\phi -\phi_{U}\right).\;$$

The boundary situations are:

(8) $$\begin{array}{ll} \chi =0,\;\theta =\theta_{L},\;\phi =\phi_{L} & \mathrm{at}\;z=0\;\mathrm{for}\;0<x<H_{x},\\ \chi =0,\;\theta =\theta_{U},\;\phi =\phi_{U} & \mathrm{at}\;z=H_{z}\;\mathrm{for}\;0<x<H_{x},\\ \psi =\partial \theta /\partial x=\partial \phi /\partial x=0 & \mathrm{at}\;x=0,\;H_{x}\;\mathrm{for}\;0<z<H_{z}. \end{array}$$

For no-dimensional examination, the dimensionless variables are described as:

(9) $$\left(\tilde{x},\tilde{z}\right)=\left(\frac{x}{H_{x}},\frac{z}{H_{z}}\right),\;\tilde{\tau }=\frac{\alpha_{E}}{\varepsilon H_{z}{}^{2}}\tau ,\;\tilde{\chi }=\frac{\chi }{\alpha_{E}},\;\tilde{\theta }=\frac{\left(\theta -\theta_{U}\right)}{\left(\theta_{L}-\theta_{U}\right)},\;\tilde{\phi }=\frac{\left(\phi -\phi_{U}\right)}{\left(\phi_{L}-\phi_{U}\right)}.$$

where $\alpha_{E}=\frac{k_{E}}{\left(\rho c\right)}$. Then, the dimensionless forms of Equations (5)–(8) become:

(10) $${\tilde{\nabla }}_{A}{}^{2}\tilde{\chi }=\left(1+\gamma \frac{\partial }{\partial \tilde{\tau }}\right)\left[-\frac{1}{Va}\frac{\partial }{\partial \tilde{\tau }}\left({\tilde{\nabla }}_{A}{}^{2}\tilde{\chi }\right)+AR_{DT}\frac{\partial \tilde{\theta }}{\partial \tilde{x}}+AR_{DS}\frac{\partial \tilde{\phi }}{\partial \tilde{x}}\right],$$

(11) $$m\frac{\partial \tilde{\theta }}{\partial \tilde{\tau }}+A\frac{\partial \left(\tilde{\chi },\tilde{\theta }\right)}{\partial \left(\tilde{x},\tilde{z}\right)}=\left({\tilde{\nabla }}_{A}{}^{2}+S_{N}\right)\tilde{\theta },$$

(12) $$\frac{\partial \tilde{\phi }}{\partial \tilde{\tau }}+A\frac{\partial \left(\tilde{\chi },\tilde{\phi }\right)}{\partial \left(\tilde{x},\tilde{z}\right)}=\left(\frac{1}{Le}{\tilde{\nabla }}_{A}{}^{2}+K_{RN}\right)\tilde{\phi },$$

(13) $$\begin{array}{l} \tilde{\chi }=0,\;\tilde{\theta }=1,\;\tilde{\phi }=1\;\;\;\;\mathrm{at}\;\tilde{z}=0\;\;\;\mathrm{for}\;0<\tilde{x}<1,\\ \tilde{\chi }=0,\;\tilde{\theta }=0,\;\tilde{\phi }=0\;\;\;\;\mathrm{at}\;\tilde{z}=1\;\;\;\mathrm{for}\;0<\tilde{x}<1,\\ \tilde{\chi }=\partial \tilde{\theta }/\partial \tilde{x}=\partial \tilde{\phi }/\partial \tilde{x}=0\;\;\mathrm{at}\;\tilde{x}=0,\;1\;\mathrm{for}\;0<\tilde{z}<1.\;\; \end{array}\}$$

Here, ${\tilde{\nabla }}_{A}{}^{2}=A^{2}\frac{\partial^{2}}{\partial {\tilde{x}}^{2}}+\frac{\partial^{2}}{\partial {\tilde{z}}^{2}}$, $\frac{\partial \left(\tilde{\chi },\tilde{\theta }\right)}{\partial \left(\tilde{x},\tilde{z}\right)}=\frac{\partial \tilde{\chi }}{\partial \tilde{x}}\frac{\partial \tilde{\theta }}{\partial \tilde{z}}-\frac{\partial \tilde{\chi }}{\partial \tilde{z}}\frac{\partial \tilde{\theta }}{\partial \tilde{x}}$, $\gamma =\frac{\gamma \alpha_{E}}{\varepsilon H_{z}{}^{2}}$ (relaxation parameter), $Va=\frac{\varepsilon^{2}\mu H_{z}{}^{2}}{\rho_{0}\alpha_{E}K}$ (Vadasz number), $A=\frac{H_{z}}{H_{x}}$ (aspect ratio), $R_{DT}=\frac{\rho_{0}g\beta_{\theta }\left(\theta_{L}-\theta_{U}\right)KH_{z}}{\mu \alpha_{E}}\;$ (thermal Rayleigh–Darcy number), $m=\frac{{\left(\rho c\right)}_{E}}{\varepsilon \left(\rho c\right)}$ (heat capacity ratio), $R_{DS}=\frac{\rho_{0}g\beta_{\phi }\left(\phi_{L}-\phi_{U}\right)KH_{z}}{\mu \alpha_{E}}\;$ (solute Rayleigh–Darcy number), $S_{N}=\frac{{SH_{z}}^{2}}{k_{E}}$ (interior heating parameter), $Le=\frac{\alpha_{E}}{\varepsilon D_{S}}$ (Lewis number), and $K_{RN}=\frac{\varepsilon K_{R}H_{z}{}^{2}}{\alpha_{E}}$ (chemical reacting parameter).

### Basic Condition

For a time-free calm solution of Equations (10)–(12), it is assumed that the temperature and solute distributions for the basic solution are:

(14) $${\tilde{\chi }}_{b}=0,\;{\tilde{\theta }}_{b}={\tilde{\theta }}_{b}\left(\tilde{z}\right),\;{\tilde{\phi }}_{b}={\tilde{\phi }}_{b}\left(\tilde{z}\right).$$

On solving the Equations (11)–(13) for the basic solution, we have:

(15) $${\tilde{\theta }}_{b}=\cos\left[\tilde{z}\sqrt{S_{N}}\right]-\cot\left[\sqrt{S_{N}}\right]\sin\left[\tilde{z}\sqrt{S_{N}}\right],$$

(16) $${\tilde{\phi }}_{b}=\cos\left[\tilde{z}\sqrt{K_{RN}Le}\right]-\cot\left[\sqrt{K_{RN}Le}\right]\sin\left[\tilde{z}\sqrt{K_{RN}Le}\right].$$

In the lack of interior heating and chemical reaction, Equations (15) and (16) give:

(17) $${\tilde{\theta }}_{b}={\tilde{\phi }}_{b}=1-\tilde{z}.$$

Equation (17) is the one found by Kuznetsov and Nield [61] for the case of pure fluid.

## 3. Perturbation Equation

Now, perturbation on the basic condition is imposed as:

(18) $$\tilde{\chi }={\tilde{\chi }}^{\prime },\tilde{\theta }={\tilde{\theta }}_{b}+\tilde{\theta^{\prime }},\;\tilde{\phi }={\tilde{\phi }}_{b}+\tilde{\phi^{\prime }}.$$

where, ${\tilde{\chi }}^{\prime }$, $\tilde{\theta^{\prime }}$, and $\tilde{\phi^{\prime }}$ are the perturbed variables on their basic estimates. Replacing Equation (18) into Equations (10)–(13), we have:

(19) $${\tilde{\nabla }}_{A}{}^{2}{\tilde{\chi }}^{\prime }=\left(1+\gamma \frac{\partial }{\partial \tilde{\tau }}\right)\left[-\frac{1}{Va}\frac{\partial }{\partial \tilde{\tau }}\left({\tilde{\nabla }}_{A}{}^{2}{\tilde{\chi }}^{\prime }\right)+AR_{DT}\frac{\partial \tilde{\theta^{\prime }}}{\partial \tilde{x}}+AR_{DS}\frac{\partial \tilde{\phi^{\prime }}}{\partial \tilde{x}}\right],$$

(20) $$m\frac{\partial \tilde{\theta^{\prime }}}{\partial \tilde{\tau }}+A\frac{\partial {\tilde{\chi }}^{\prime }}{\partial \tilde{x}}\frac{\partial {\tilde{\theta }}_{b}}{\partial \tilde{z}}+A\frac{\partial \left({\tilde{\chi }}^{\prime },\tilde{\theta^{\prime }}\right)}{\partial \left(\tilde{x},\tilde{z}\right)}=\left({\tilde{\nabla }}_{A}{}^{2}+S_{N}\right)\tilde{\theta^{\prime }},\;$$

(21) $$\frac{\partial \tilde{\phi^{\prime }}}{\partial \tilde{\tau }}+A\frac{\partial {\tilde{\chi }}^{\prime }}{\partial \tilde{x}}\frac{\partial {\tilde{\phi }}_{b}}{\partial \tilde{z}}+A\frac{\partial \left({\tilde{\chi }}^{\prime },\tilde{\phi^{\prime }}\right)}{\partial \left(\tilde{x},\tilde{z}\right)}=\left(\frac{1}{Le}{\tilde{\nabla }}_{A}{}^{2}+K_{RN}\right)\tilde{\phi^{\prime }},$$

(22) $$\begin{array}{l} {\tilde{\chi }}^{\prime }=\tilde{\theta^{\prime }}=\tilde{\phi^{\prime }}=0\;\;\;\;\mathrm{at}\;\tilde{z}=0,1\;\;\;\;\mathrm{for}\;0<\tilde{x}<1,\\ {\tilde{\chi }}^{\prime }=\partial \tilde{\theta^{\prime }}/\partial \tilde{x}=\partial \tilde{\phi^{\prime }}/\partial \tilde{x}=0\;\;\;\mathrm{at}\;\tilde{x}=0,\;1\;\;\;\mathrm{for}\;0<\tilde{z}<1. \end{array}$$

## 4. Linear Stability Consideration

In this segment, the thresholds of the marginal and oscillatory type of convective flows are determined applying linear theory. Now, it is supposed that the amplitudes of the perturbation are extremely small and expressed as [13,29,60,62]:

(23) $${\tilde{\chi }}^{\prime }=\bar{\chi }\left(\tilde{z}\right)e^{i\sigma \tilde{\tau }}\cos\lambda \tilde{x},\;\tilde{\theta^{\prime }}=\bar{\theta }\left(\tilde{z}\right)e^{i\sigma \tilde{\tau }}\sin\lambda \tilde{x},\tilde{\phi^{\prime }}=\bar{\phi }\left(\tilde{z}\right)e^{i\sigma \tilde{\tau }}\sin\lambda \tilde{x}.$$

where λ and σ represent the wavenumber and enlargement rate of disturbances, correspondingly.

On using Equation (23) in Equations (19)–(22) and avoiding the nonlinear terms with perturbed variables, we have:

(24) $$\left(D^{2}-A^{2}\lambda^{2}\right)\bar{\chi }-\left(1+i\gamma \sigma \right)\left[-\frac{i\sigma }{Va}\left(D^{2}-A^{2}\lambda^{2}\right)\bar{\chi }+A\lambda R_{DT}\bar{\theta }+A\lambda R_{DS}\bar{\phi }\right]=0,$$

(25) $$\lambda A\bar{\chi }D{\tilde{\theta }}_{b}+\left(D^{2}-A^{2}\lambda^{2}+S_{N}-i\sigma m\right)\bar{\theta }=0,$$

(26) $$\lambda A\bar{\chi }D{\tilde{\phi }}_{b}+\left[\frac{1}{Le}\left(D^{2}-A^{2}\lambda^{2}\right)+K_{RN}-i\sigma \right]\bar{\phi }=0,$$

(27) $$\bar{\chi }=\bar{\theta }=\bar{\phi }=0\;\;\;\;\mathrm{at}\;\tilde{z}=0,1\;\;\;\;\mathrm{for}\;0<\tilde{x}<1.$$

Here, $\frac{d}{d\tilde{z}}\equiv D$. To find an approximate solution to the system of Equations (24)–(27), the Galerkin routine is utilized [63,64,65,66,67,68]. The trial functions (fulfilling the boundary circumstances) are picked as:

(28) $$\bar{\chi }=F\sin\pi \tilde{z},\;\bar{\theta }=E\sin\pi \tilde{z},\;\bar{\phi }=G\sin\pi \tilde{z}.\;$$

where, E, F, and G are unidentified coefficients. On applying Equation (28) into Equations (24)–(26) and for the non-singular solution, we have:

(29) $$\left|\begin{array}{lll} \frac{Q_{1}\left(-i\sigma +\gamma \sigma^{2}-Va\right)}{2Va} & \frac{1}{2}\lambda AR_{DT}\left(-1-i\gamma \sigma \right) & \frac{1}{2}\lambda AR_{DS}\left(-1-i\gamma \sigma \right)\\ Q_{2} & \frac{1}{2}\left(S_{N}-Q_{1}-im\sigma \right) & \;0\\ Q_{3} & \;0\; & -\frac{\left(Q_{1}+i\sigma Le-K_{RN}Le\right)}{2Le} \end{array}\right|=0.$$

where, $Q_{1}=\pi^{2}+\lambda^{2}A^{2}$, $Q_{2}=\frac{2\lambda A\pi^{2}}{\left(S_{N}-4\pi^{2}\right)}$ and $Q_{3}=\frac{2\lambda A\pi^{2}}{\left(K_{RN}Le-4\pi^{2}\right)}$.

Now, from Equation (25), we have:

(30) $$R_{DT}=N_{1}+i\sigma N_{2}.$$

where,

(31) $$\begin{array}{ll} N_{1} & =-\frac{LeQ_{3}R_{DS}\left[Q_{1}\left(Q_{1}-S_{N}\right)+Le\left\{m\sigma^{2}+K_{RN}\left(S_{N}-Q_{1}\right)\right\}\right]}{\left[Le^{2}\left(K_{RN}{}^{2}+\sigma^{2}\right)-2K_{RN}LeQ_{1}+Q_{1}{}^{2}\right]Q_{2}}\\ & +\frac{m\sigma^{2}Q_{1}+\gamma^{2}m\sigma^{4}Q_{1}-\gamma m\sigma^{2}Q_{1}Va-Q_{1}{}^{2}Va+Q_{1}S_{N}Va}{2\lambda AQ_{2}Va+2\lambda A\sigma^{2}\gamma^{2}Q_{2}Va}, \end{array}$$

(32) $$\begin{array}{ll} N_{2} & =\frac{LeQ_{3}R_{DS}\left[K_{RN}Lem-mQ_{1}+Le\left(Q_{1}-S_{N}\right)\right]}{\left[Le^{2}\left(K_{RN}{}^{2}+\sigma^{2}\right)-2K_{RN}LeQ_{1}+Q_{1}{}^{2}\right]Q_{2}}\\ & +\frac{Q_{1}\left\{\gamma \left(Q_{1}-S_{N}\right)-m\right\}Va-Q_{1}\left(Q_{1}-S_{N}\right)\left(1+\gamma^{2}\sigma^{2}\right)}{2\lambda A\left(1+\gamma^{2}\sigma^{2}\right)Q_{2}Va}. \end{array}$$

### 4.1. Marginal Pattern of Convection

The marginal pattern of convection can happen, if σ=0. Thus, Equation (30) gives the marginal thermal Rayleigh–Darcy number $R_{DT}^{M}$ as:

(33) $$R_{DT}^{M}=\frac{\left(\lambda^{2}A^{2}+\pi^{2}\right)\left(\lambda^{2}A^{2}+\pi^{2}-S_{N}\right)\left(4\pi^{2}-S_{N}\right)}{4\lambda^{2}A^{2}\pi^{2}}-\frac{LeR_{DS}\left(\lambda^{2}A^{2}+\pi^{2}-S_{N}\right)\left(4\pi^{2}-S_{N}\right)}{\left(\lambda^{2}A^{2}+\pi^{2}-K_{RN}Le\right)\left(4\pi^{2}-K_{RN}Le\right)}.$$

The threshold of the $R_{DT}^{M}$ signifying the beginning of marginal convection occurs at $\lambda_{c}$ where $\lambda_{c}=\sqrt{a}$ satisfies the equation:

(34) $$\begin{array}{l} A^{8}\left(K_{RN}Le-4\pi^{2}\right)a^{4}-2A^{6}\left(K_{RN}Le-4\pi^{2}\right)\left(K_{RN}Le-\pi^{2}\right)a^{3}+A^{4}\{K_{RN}Le\\ \left(K_{RN}{}^{2}Le^{2}-6K_{RN}Le\pi^{2}+8\pi^{4}-4\pi^{2}LeR_{DS}\right)+\pi^{2}\left(K_{RN}Le-4\pi^{2}+4LeR_{DS}\right)S_{N}\}a^{2}\\ +2A^{2}\pi^{2}\left(\pi^{2}-K_{RN}Le\right)\left(4\pi^{2}-K_{RN}Le\right)\left(\pi^{2}-S_{N}\right)a+\pi^{2}\left(4\pi^{2}-K_{RN}Le\right)\\ {\left(\pi^{2}-K_{RN}Le\right)}^{2}\left(\pi^{2}-S_{N}\right)=0. \end{array}$$

In the nonattendance of chemical response ($K_{RN}\to 0$), Equations (33) and (34) turn into:

(35) $$R_{DT}^{M}=\frac{\left(\lambda^{2}A^{2}+\pi^{2}\right)\left(\lambda^{2}A^{2}+\pi^{2}-S_{N}\right)\left(4\pi^{2}-S_{N}\right)}{4\lambda^{2}A^{2}\pi^{2}}-\frac{LeR_{DS}\left(\lambda^{2}A^{2}+\pi^{2}-S_{N}\right)\left(4\pi^{2}-S_{N}\right)}{4\pi^{2}\left(\lambda^{2}A^{2}+\pi^{2}\right)},$$

(36) $$A^{8}a^{4}+2A^{6}\pi^{2}a^{3}+A^{4}S_{N}\left(\pi^{2}-LeR_{DS}\right)a^{2}+2A^{2}\pi^{4}\left(S_{N}-\pi^{2}\right)a-\pi^{8}+\pi^{6}S_{N}=0.$$

For the case of a single component ($R_{DS}\to 0$), Equations (35) and (36) give:

(37) $$R_{DT}^{M}=\frac{\left(\lambda^{2}A^{2}+\pi^{2}\right)\left(\lambda^{2}A^{2}+\pi^{2}-S_{N}\right)\left(4\pi^{2}-S_{N}\right)}{4\lambda^{2}A^{2}\pi^{2}},$$

(38) $$\lambda_{c}=\frac{\sqrt{\pi \sqrt{\left(\pi^{2}-S_{N}\right)}}}{A}.$$

The Equations (37) and (38) are the same as found by Yadav and Maqhusi [29] for the lack of viscosity variation.

In the nonattendance of interior heating ($S_{N}\to 0$), Equations (35) and (36) provide:

(39) $$R_{DT}^{M}=\frac{{\left(\lambda^{2}A^{2}+\pi^{2}\right)}^{2}}{\lambda^{2}A^{2}}-LeR_{DS},$$

(40) $$\lambda_{c}=\frac{\pi }{A}.$$

Equations (39) and (40) are the standard results for a dual diffusive convective motion in a permeable medium for cases A=1 [13,50,69].

For the case of one component ($R_{DS}\to 0$), Equations (39) and (40) offer the critical marginal thermal Rayleigh–Darcy number $R_{DT,c}^{M}$ as:

(41) $$R_{DT,c}^{M}=4\pi^{2}.$$

This coincides with the conclusion of Nield and Kuznetsov [70] for the convection in a rectangular box. Additionally, Equation (41) agrees with the experimental results obtained by Horton and Rogers [71] and Katto and Masuoka [72].

### 4.2. Oscillatory Pattern of Convection

The oscillatory pattern of convective motion occurs when σ≠0 and $N_{2}=0$. Then, Equation (30) proposes the oscillatory thermal Rayleigh–Darcy number $R_{DT}^{Os}$ as:

(42) $$\begin{array}{ll} R_{DT}^{os} & =-\frac{LeQ_{3}R_{DS}\left[Q_{1}\left(Q_{1}-S_{N}\right)+Le\left\{m\sigma^{2}+K_{RN}\left(S_{N}-Q_{1}\right)\right\}\right]}{\left[Le^{2}\left(K_{RN}{}^{2}+\sigma^{2}\right)-2K_{RN}LeQ_{1}+Q_{1}{}^{2}\right]Q_{2}}\\ & +\frac{m\sigma^{2}Q_{1}+\gamma^{2}m\sigma^{4}Q_{1}-\gamma m\sigma^{2}Q_{1}Va-Q_{1}{}^{2}Va+Q_{1}S_{N}Va}{2\lambda AQ_{2}Va+2\lambda A\sigma^{2}\gamma^{2}Q_{2}Va}, \end{array}$$

Additionally, from Equation (30), the rate of oscillation σ satisfies the following dispersion relation:

(43) $$\beta_{1}{\left(\sigma^{2}\right)}^{2}+\beta_{2}\left(\sigma^{2}\right)+\beta_{3}=0.$$

Here,

$$\beta_{1}=\gamma^{2}Le^{2}Q_{1}Q_{2}\left(S_{N}-Q_{1}\right),$$

$$\begin{array}{ll} \beta_{2} & =2\lambda A\gamma^{2}LeQ_{2}Q_{3}R_{DS}\{K_{RN}Lem-mQ_{1}+Le\left(Q_{1}-S_{N}\right)\}Va+Q_{1}Q_{2}[-\{(1+\gamma^{2}K_{RN}{}^{2})Le^{2}\\ & -2\gamma^{2}K_{RN}LeQ_{1}+\gamma^{2}Q_{1}{}^{2}\}\left(Q_{1}-S_{N}\right)-Le^{2}\left\{m+\gamma \left(S_{N}-Q_{1}\right)\right\}Va], \end{array}.$$

$$\begin{array}{ll} \beta_{3} & =Q_{1}Q_{2}{\left(Q_{1}-K_{RN}Le\right)}^{2}\left\{S_{N}-\left(m+S_{N}\gamma \right)Va+Q_{1}\left(\gamma Va-1\right)\right\}\\ & +2\lambda ALeQ_{2}Q_{3}R_{DS}\{K_{RN}Lem-mQ_{1}+Le\left(Q_{1}-S_{N}\right)\}Va \end{array}.$$

From Equation (43), it is clear that the oscillatory manner of convective flow is conceivable only if $\sigma^{2}>0$. The analytical appearance for $R_{DT}^{Os}$ specified by Equation (42) is minimized respecting the wave number λ numerically when $\sigma^{2}>0$ for diverse estimates of involved physical parameters to identify their impacts on the start of the oscillatory type of convective motion.

## 5. Weak Nonlinear Stability Investigation

The linear stability inquiry offers the threshold for the start of convective movement of the Maxwell fluid in terms of $R_{DT,c}$ but does not calculate the convective heat and mass transport. To get these extra details, here the weak nonlinear stability analysis is used as [17,44,56,60,73]:

(44) $${\tilde{\chi }}^{\prime }=B_{11}\left(\tilde{\tau }\right)\sin\left(\lambda \tilde{x}\right)\sin\left(\pi \tilde{z}\right),$$

(45) $$\tilde{\theta^{\prime }}=C_{11}\left(\tilde{\tau }\right)\cos\left(\lambda \tilde{x}\right)\sin\left(\pi \tilde{z}\right)+C_{02}\left(\tilde{\tau }\right)\sin\left(2\pi \tilde{z}\right),$$

(46) $$\tilde{\phi^{\prime }}=D_{11}\left(\tilde{\tau }\right)\cos\left(\lambda \tilde{x}\right)\sin\left(\pi \tilde{z}\right)+D_{02}\left(\tilde{\tau }\right)\sin\left(2\pi \tilde{z}\right).$$

where, $B_{11}\left(\tilde{\tau }\right),\;C_{11}\left(\tilde{\tau }\right),\;C_{02}\left(\tilde{\tau }\right),\;D_{11}\left(\tilde{\tau }\right)$, and $D_{02}\left(\tilde{\tau }\right)$ are undetermined amplitudes and to be determined. On replacing Equations (44)–(46) into Equations (19)–(21), we obtain the nonlinear stability equations as:

(47) $$\frac{d^{2}B_{11}}{d{\tilde{\tau }}^{2}}=-\frac{1}{\gamma }\frac{dB_{11}}{d\tilde{\tau }}+\frac{Va}{Q_{1}\gamma }[-Q_{1}B_{11}+\lambda A(R_{DT}C_{11}+R_{DT}\gamma \frac{dC_{11}}{d\tilde{\tau }}+R_{DS}D_{11}+R_{DS}\gamma \frac{dD_{11}}{d\tilde{\tau }})],$$

(48) $$\frac{dC_{11}}{d\tilde{\tau }}=\frac{\lambda A\pi B_{11}C_{02}-2Q_{2}B_{11}-\left(Q_{1}-S_{N}\right)C_{11}}{m},$$

(49) $$\frac{dC_{02}}{d\tilde{\tau }}=\frac{\lambda A\pi \left[4\pi C_{02}-Q_{2}B_{11}C_{11}\right]}{2mQ_{2}},$$

(50) $$\frac{dD_{11}}{d\tilde{\tau }}=\lambda A\pi B_{11}D_{02}-2Q_{3}B_{11}+K_{RN}D_{11}-\frac{Q_{1}D_{11}}{Le},$$

(51) $$\frac{dD_{02}}{d\tilde{\tau }}=\frac{\lambda A\pi \left[4\pi D_{02}-Q_{3}LeB_{11}D_{11}\right]}{2Q_{3}Le},$$

where $Q_{1}=\pi^{2}+\lambda^{2}A^{2}$, $Q_{2}=\frac{2\lambda A\pi^{2}}{\left(S_{N}-4\pi^{2}\right)}$ and $Q_{3}=\frac{2\lambda A\pi^{2}}{\left(K_{RN}Le-4\pi^{2}\right)}$.

The above nonlinear equations are not fit for analytical inspection for the time-reliant variables. Thus, we solved it numerically utilizing the Runge–Kutta–Fehlberg technique (RKF45). The results are also validated with ODE45 solver in MATLAB. For the initial state, we select $B_{11}=1,\;C_{11}=0,\;C_{02}=0,\;D_{11}=0$, and $D_{02}=0$.

### 5.1. Steady Motion

For steady motion, Equations (47)–(51) become:

(52) $$B_{11}=\frac{\lambda A}{Q_{1}}\left[R_{DT}C_{11}+R_{DS}D_{11}\right],\;$$

(53) $$\lambda A\pi B_{11}C_{02}-2Q_{2}B_{11}-\left(Q_{1}-S_{N}\right)C_{11}=0,$$

(54) $$4\pi C_{02}-Q_{2}B_{11}C_{11}=0,$$

(55) $$\lambda A\pi B_{11}D_{02}-2Q_{3}B_{11}+K_{RN}D_{11}-\frac{Q_{1}D_{11}}{Le}=0,$$

(56) $$4\pi D_{02}-Q_{3}LeB_{11}D_{11}=0.$$

On solving Equations (52)–(56) analytically, we have:

(57) $$B_{11}=\sqrt{\frac{-Q_{5}+\sqrt{Q_{5}{}^{2}-4Q_{4}Q_{6}}}{2Q_{4}}},$$

(58) $$C_{11}=\frac{8Q_{2}B_{11}}{4S_{N}-4Q_{1}+\lambda AQ_{2}B_{11}{}^{2}},$$

(59) $$C_{02}=\frac{2Q_{2}{}^{2}B_{11}{}^{2}}{4\pi S_{N}-4\pi Q_{1}+\lambda A\pi Q_{2}B_{11}{}^{2}},$$

(60) $$D_{11}=\frac{8LeQ_{3}B_{11}}{4K_{RN}Le-4Q_{1}+\lambda ALe^{2}Q_{3}B_{11}{}^{2}},$$

(61) $$D_{02}=\frac{2Le^{2}Q_{3}{}^{2}B_{11}{}^{2}}{4\pi K_{RN}Le-4\pi Q_{1}+\lambda A\pi Le^{2}Q_{3}B_{11}{}^{2}}.$$

Here,

$$Q_{1}=\pi^{2}+\lambda^{2}A^{2},\;Q_{2}=\frac{2\lambda A\pi^{2}}{\left(S_{N}-4\pi^{2}\right)},\;Q_{3}=\frac{2\lambda A\pi^{2}}{\left(K_{RN}Le-4\pi^{2}\right)},\;Q_{4}=\lambda^{2}A^{2}Le^{2}Q_{1}Q_{2}Q_{3},$$

$$Q_{5}=4\lambda A\left\{K_{RN}LeQ_{1}Q_{2}-Q_{1}{}^{2}\left(Q_{2}+Le^{2}Q_{3}\right)-2\lambda ALeQ_{2}Q_{3}\left(R_{DS}+R_{DT}Le\right)+Le^{2}Q_{1}Q_{3}S_{N}\right\},$$

$$\begin{array}{ll} Q_{6}= & -16\{-Q_{1}{}^{3}-2\lambda AQ_{1}\left(LeQ_{3}R_{DS}+Q_{2}R_{DT}\right)+Q_{1}{}^{2}S_{N}+2\lambda ALeQ_{3}R_{DS}S_{N}\\ & +K_{RN}Le\left(Q_{1}{}^{2}+2\lambda AQ_{2}R_{DT}-Q_{1}S_{N}\right)\;\}. \end{array}$$

### 5.2. Convective Heat and Mass Transports

Convective heat and mass transfers play a very vital role in detecting the convective motion in more early stages. Heat and mass transfers can be calculated in spans of Nusselt number Nu and Sherwood number Sh, individually, and described as [17,44,56,60,73]:

(62) $$Nu\left(\tilde{\tau }\right)=1+{\left[\int_{0}^{2\pi /\lambda }\left(\frac{\partial \tilde{\theta^{\prime }}}{\partial \tilde{z}}\right)d\tilde{x}/\int_{0}^{2\pi /\lambda }\left(\frac{\partial {\tilde{\theta }}_{b}}{\partial \tilde{z}}\right)d\tilde{x}\right]}_{\tilde{z}=0},$$

(63) $$Sh\left(\tilde{\tau }\right)=1+{\left[\int_{0}^{2\pi /\lambda }\left(\frac{\partial \tilde{\phi^{\prime }}}{\partial \tilde{z}}\right)d\tilde{x}/\int_{0}^{2\pi /\lambda }\left(\frac{\partial {\tilde{\phi }}_{b}}{\partial \tilde{z}}\right)d\tilde{x}\right]}_{\tilde{z}=0}.$$

On applying Equations (15), (16), (45) and (46) into Equations (62) and (63), we have:

(64) $$Nu\left(\tilde{\tau }\right)=1-\frac{2\pi C_{02}\left(\tilde{\tau }\right)\tan\left[\sqrt{S_{N}}\right]}{\sqrt{S_{N}}},$$

(65) $$Sh\left(\tilde{\tau }\right)=1-\frac{2\pi D_{02}\left(\tilde{\tau }\right)\tan\left[\sqrt{K_{RN}Le}\right]}{\sqrt{K_{RN}Le}}.$$

## 6. Results and Discussion

**Figure 2:**
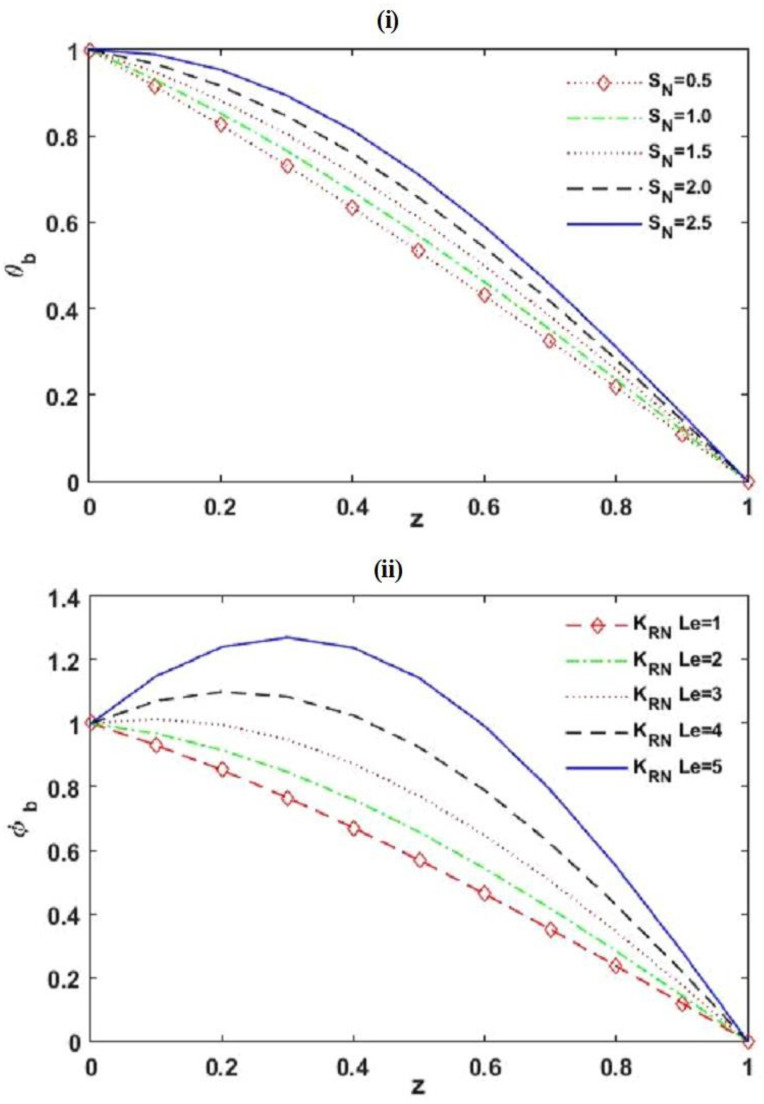
Distributions of basic state temperature (Le) and solute concentration (${\tilde{\phi }}_{b}$ ) with the depth of Maxwell fluid layer $\tilde{z}$.

**Figure 3:**
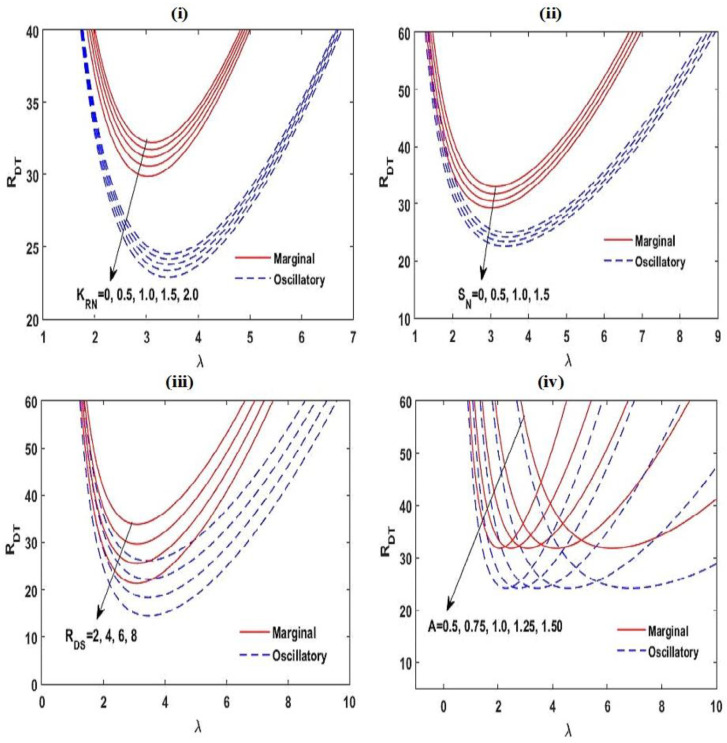
The effect of $K_{RN}$, $S_{N}$, $R_{DS}$, and A on the neutral stability curves at Le=2, γ=0.5, m=1.5, A=1, $R_{DS}=3$, $S_{N}=0.5$, $K_{RN}=0.5$, and Va=3 with variation in one of these parameters.

**Figure 4:**
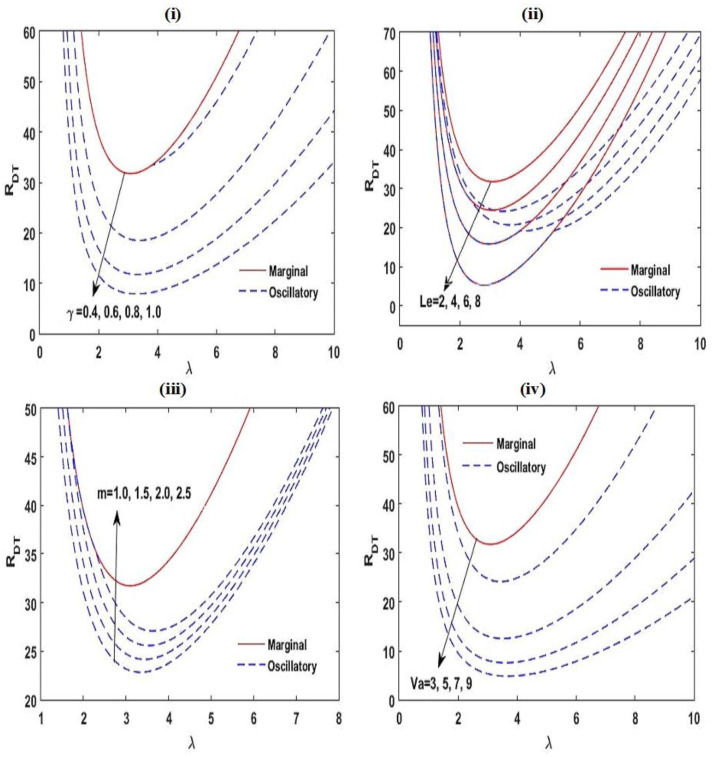
The effect of γ, Le, m, and Va on the neutral stability curves at Le=2, γ=0.5, m=1.5, A=1, $R_{DS}=3$, $S_{N}=0.5$, $K_{RN}=0.5$, and Va=3 with variation in one of these parameters.

**Figure 5:**
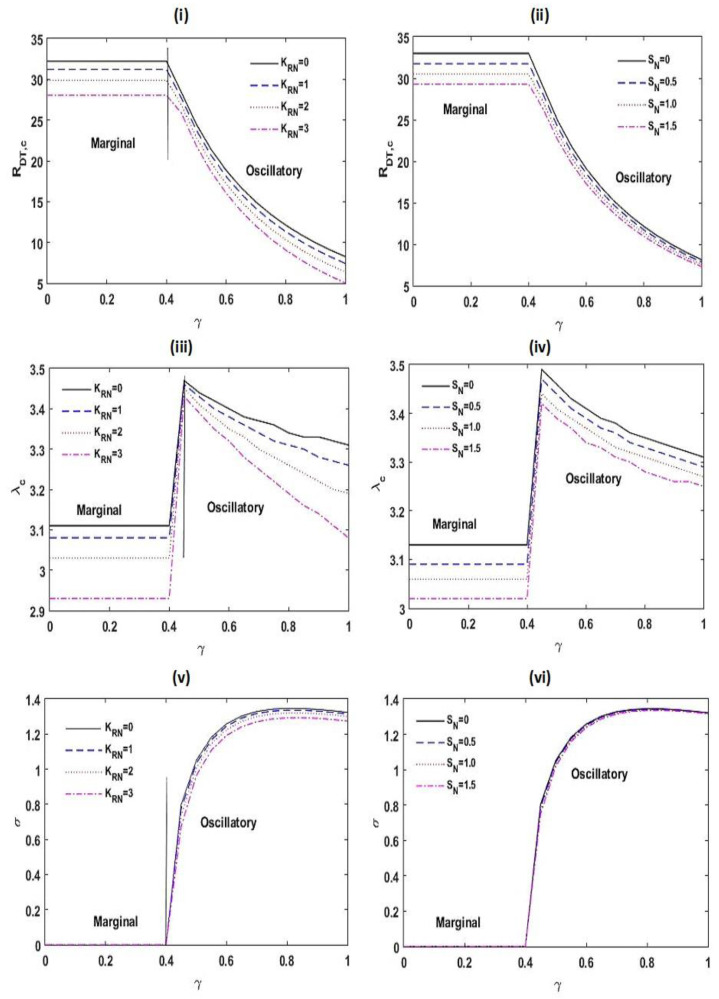
Variation in $R_{DT,c}$, $\lambda_{c}$, and σ with γ for different values of $K_{RN}$ and $S_{N}$ at Le=2, m=1.5, A=1, $R_{DS}=3$, $S_{N}=0.5$, $K_{RN}=0.5$, and Va=3.

**Figure 6:**
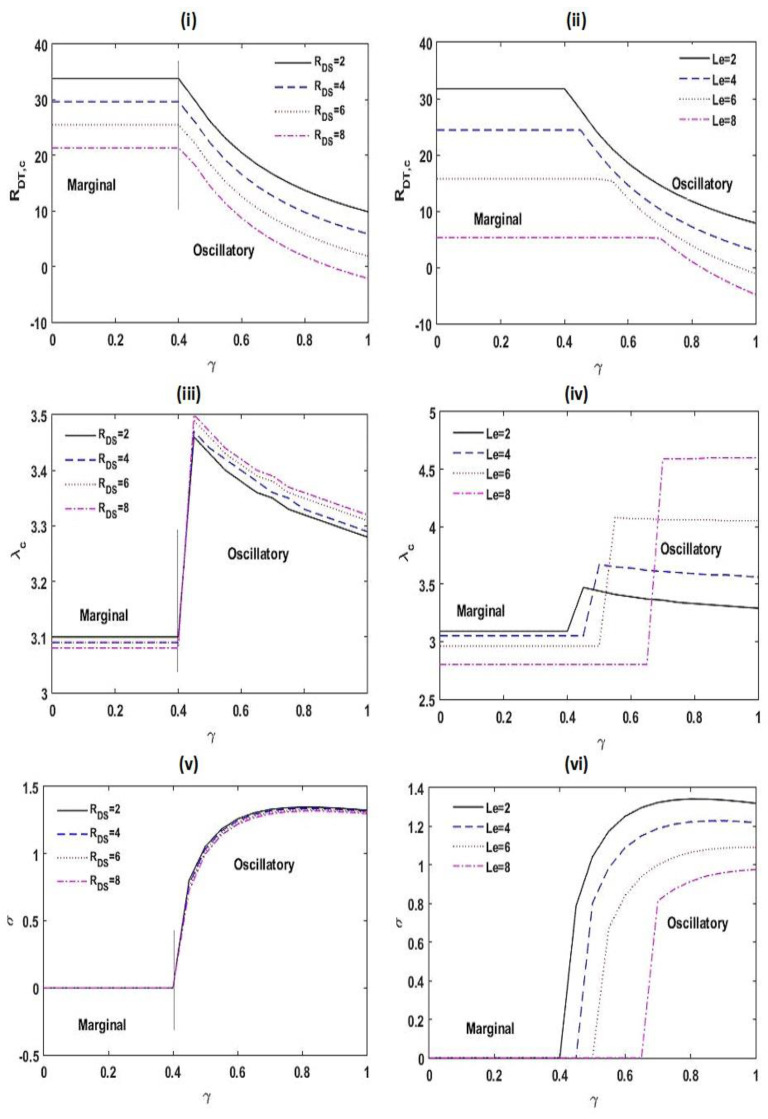
Variation in $R_{\mathrm{DT},c}$, $\lambda_{c}$, and σ with γ for different values of $R_{DS}$ and Le at Le=2, m=1.5, A=1, $R_{DS}=3$, $S_{N}=0.5$, $K_{RN}=0.5$, and Va=3.

**Figure 7:**
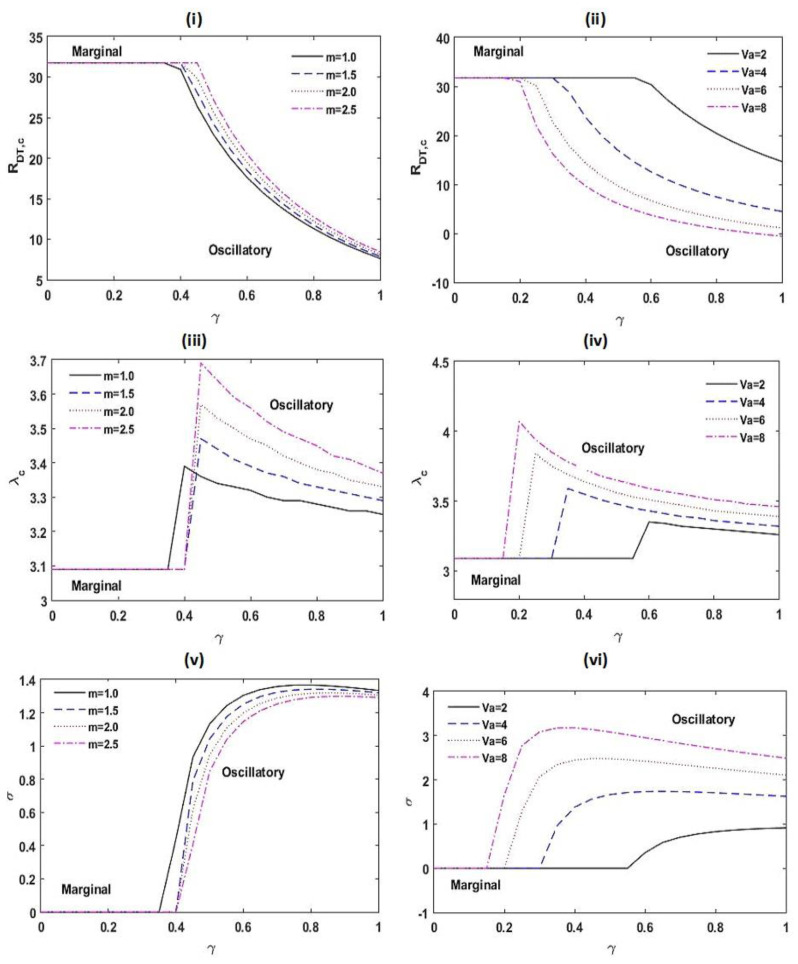
Variation in $R_{\mathrm{DT},c}$, $\lambda_{c}$, and σ with γ for different values of m and Va at Le=2, m=1.5, A=1, $R_{DS}=3$, $S_{N}=0.5$, $K_{RN}=0.5$, and Va=3.

**Figure 8:**
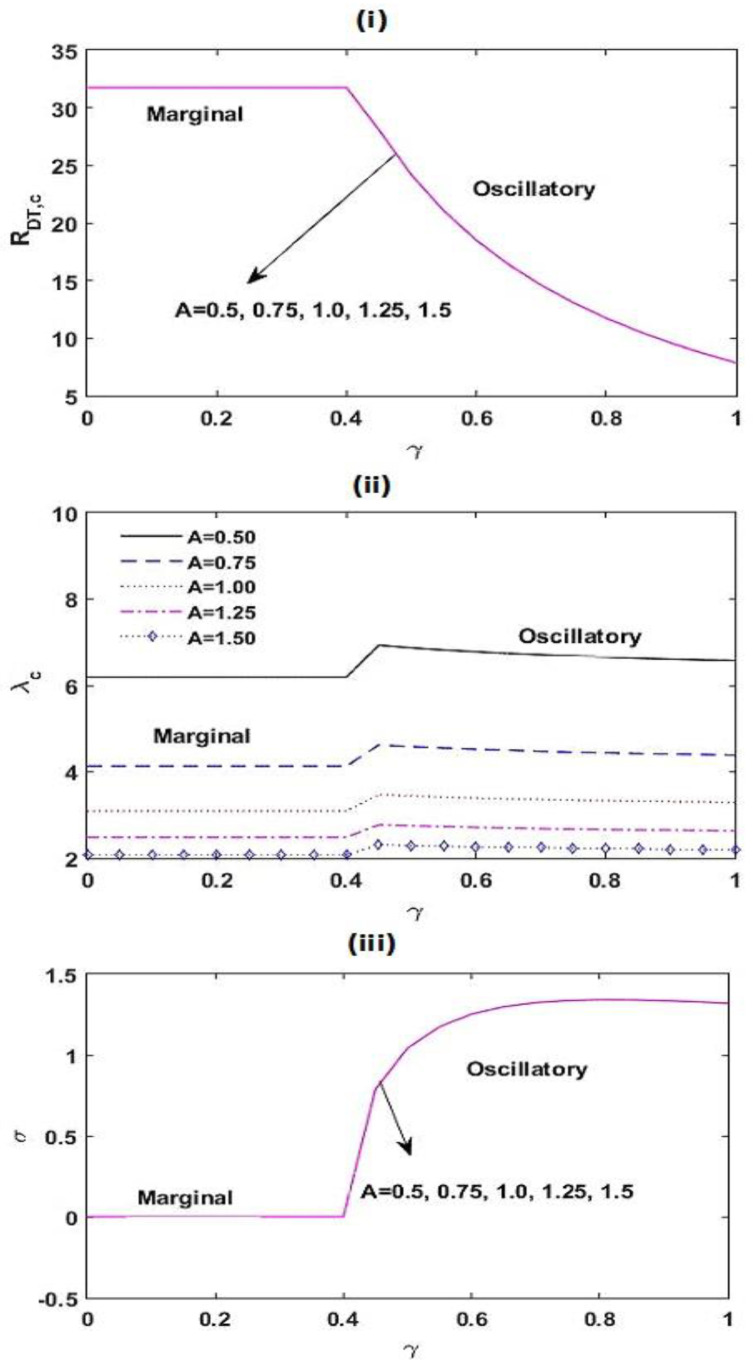
Variation in $R_{DT,c}$, $\lambda_{c}$, and σ with γ for different values of A at Le=2, m=1.5, A=1, $R_{DS}=3$, $S_{N}=0.5$, $K_{RN}=0.5$, and Va=3.

**Figure 9:**
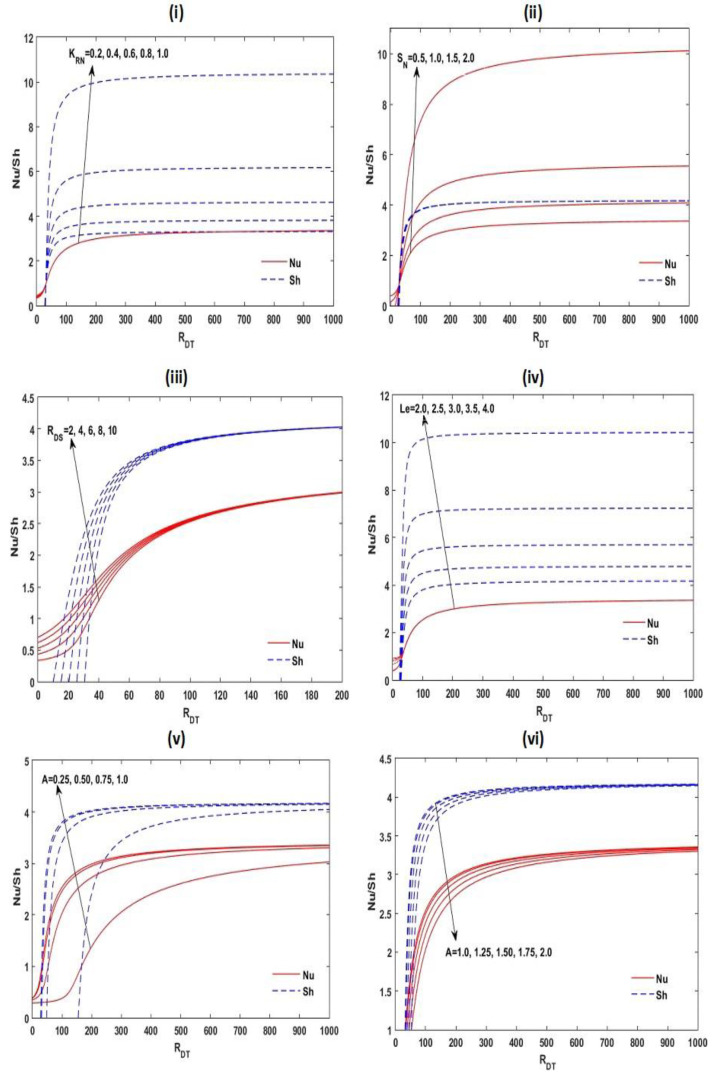
Disparity of the steady Nu and Sh with $R_{\mathrm{DT}}$ at Le=2, A=1, $R_{DS}=3$, $S_{N}=0.5$, and $K_{RN}=0.5$ for different values of one of these parameters.

**Figure 10:**
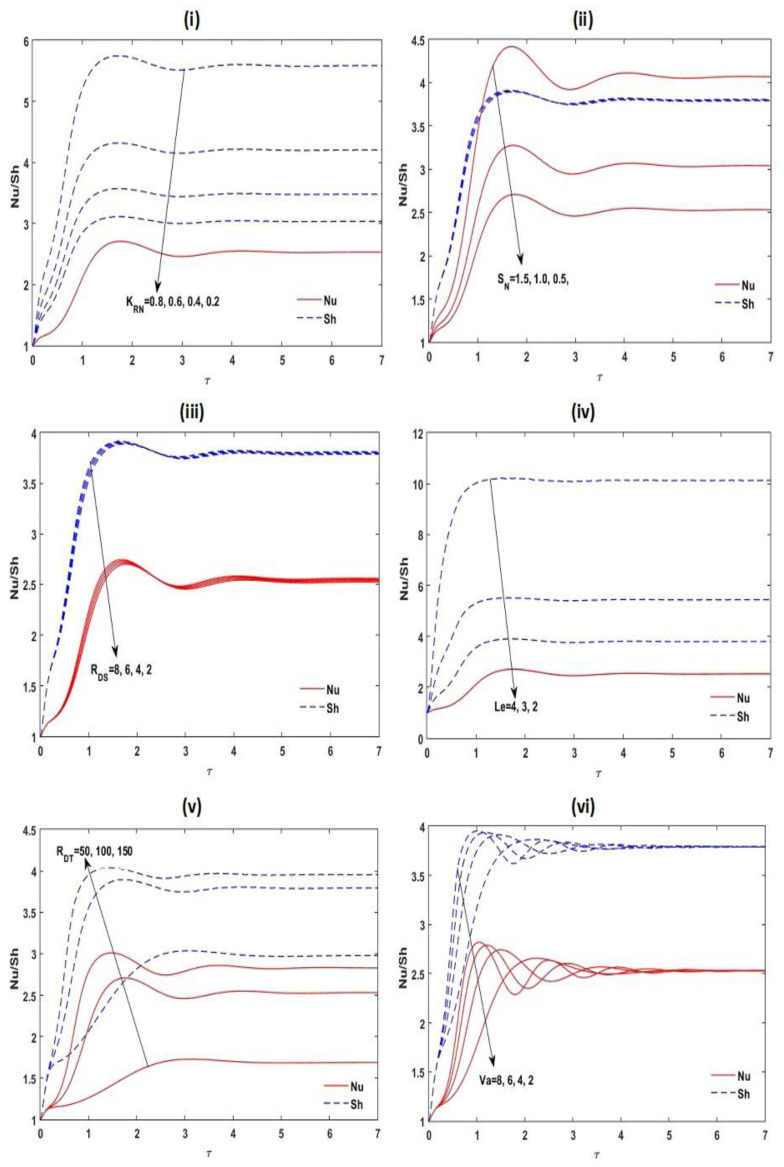
Disparity of the unsteady Nu and Sh with $\tilde{\tau }$ for different values of $K_{RN}$, $K_{RN}$, $R_{DS}$, Le, $R_{DT}$, and Va at Le=2, m=1.5, A=1, $R_{DS}=3$, $S_{N}=0.5$, γ=0.5,$K_{RN}=0.5$, and Va=3.

**Figure 11:**
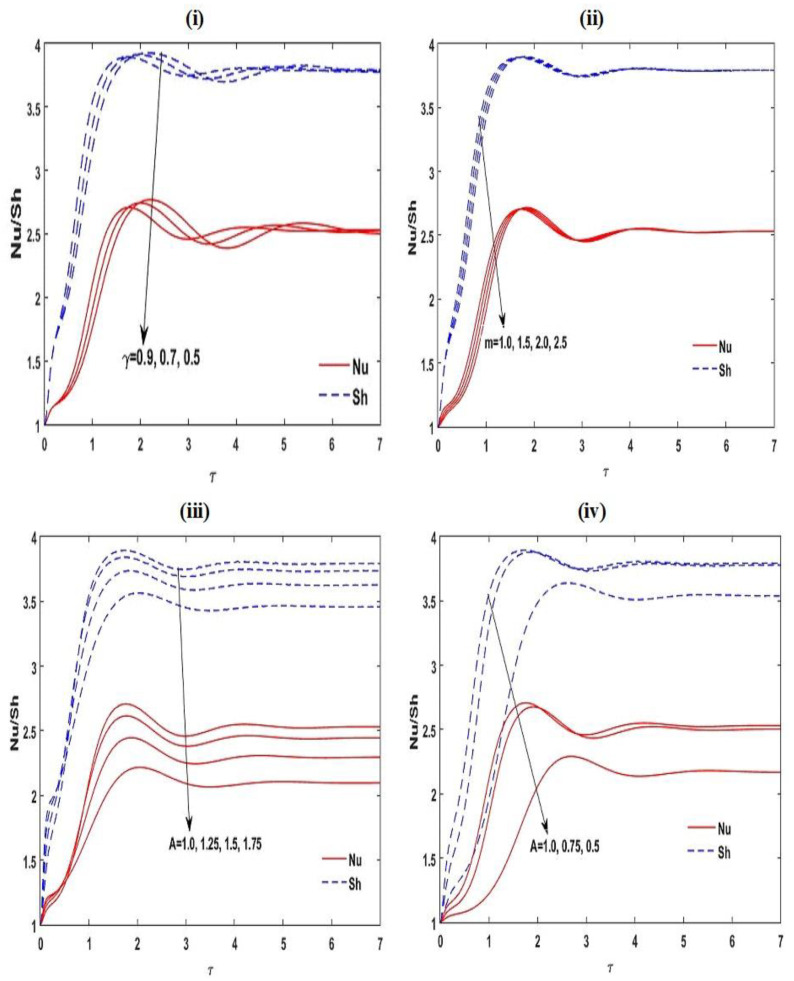
Disparity of the unsteady Nu and Sh with $\tilde{\tau }$ for different values of γ, m, and A at Le=2, m=1.5, A=1, $R_{DS}=3$, $S_{N}=0.5$, γ=0.5,$K_{RN}=0.5$, and Va=3.

**Figure 12:**
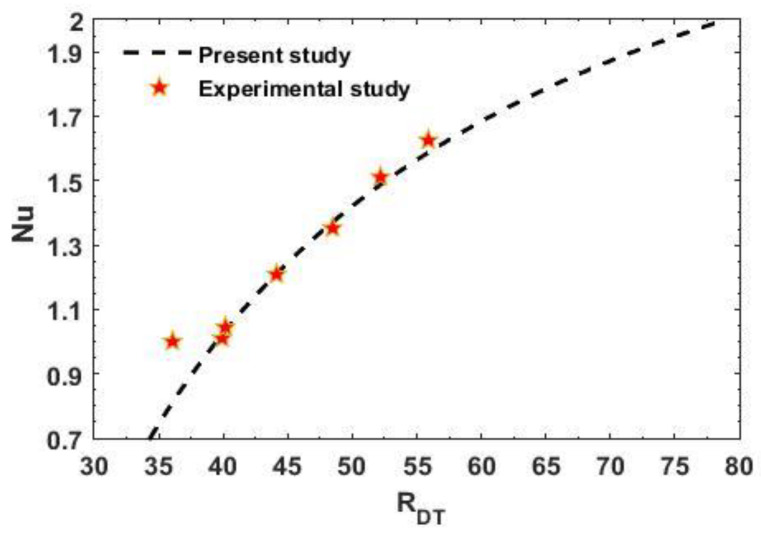
Contrast of the Nusselt number Nu in the particular case of the present analysis with experimental results found by Serkitjis [77].

The impact of interior heating and chemical reaction on the beginning of dual diffusive convective motion and the convective heat and mass transfers in non-Newtonian Maxwell fluid-saturated permeable square (A=1), slender (A<1), and rectangular (A>1) enclosures were explored. Employing the linear stability philosophy, the criteria for the start of the marginal and oscillatory pattern of convective motions were derived analytically in terms of $R_{DT,c}$, which is the function of physical parameters $K_{RN},\;S_{N},\;R_{DS},\;Le,\;m,\;Va,\;A$, and γ. Applying the weakly nonlinear theory, the convective heat and mass transports were calculated in spans of the Nusselt number Nu and Sherwood number Sh, individually. The results are presented in Figure 2, Figure 3, Figure 4, Figure 5, Figure 6, Figure 7, Figure 8, Figure 9, Figure 10, Figure 11 and Figure 12 and Table 1, Table 2, Table 3 and Table 4. To create the numerical results and figures, the MATLAB software (R2018b) was used. The range of the physical parameters that were considered for making the results are given in the figure captions and obtained from the available literature [31,50,55,60,73,74,75].

Figure 2 illustrates the impact of $S_{N}$ on the distribution of basic state temperature ${\tilde{\theta }}_{b}$, and the impact of $K_{RN}$ and Le on the allocation of basic state solute concentration ${\tilde{\phi }}_{b}$. From Figure 2i, it is recognized that the power of the basic temperature allocation increases with accumulating interior heating parameters $S_{N}$ and the profile of basic temperature allocation alters from linear to nonlinear with $S_{N}$. This happened because the increasing $S_{N}$ provides more warming to the Maxwell fluid layer, which enhances the strength of buoyancy force and as a result, more disturbances are seen in the system. From Figure 2ii, we observed a similar result on the allocation of basic state solute concentration ${\tilde{\phi }}_{b}$ with increasing $K_{RN}$ and Le. This happened because an increase in the strength of $K_{RN}$ and Le creates more disturbances in the system.

Figure 3 and Figure 4 exhibit the neutral curves for diverse values of the chemical reacting parameter $K_{RN}$ (Figure 3i), the interior heating parameter $S_{N}$ (Figure 3ii), the solute Rayleigh–Darcy number $R_{DS}$ (Figure 3iii), the aspect ratio A (Figure 3iv), the relaxation parameter γ (Figure 4i), the Lewis number Le (Figure 4ii), the heat capacity ratio m (Figure 4iii), and the Vadasz number Va (Figure 4iv). From these plots, it is found that the neutral curves are linked in a topological way. This establishes that the linear stability of the arrangement is specified in the span of $R_{DT,c}$, which at lower values the arrangement is stable and at $R_{DT}$ somewhat greater than $R_{DT,c}$, convective activity starts. From these figures, it is also established that by increasing the values of $K_{RN}$, $S_{N},$$R_{DS}$, γ, Le, and Va, the estimate of $R_{DT,c}$ tends to lessen, i.e., the arrangement goes to destabilize, while m has a stabilizing impact on the stability of the structure. The aspect ratio A does not affect $R_{DT,c}$. The marginal pattern of the convective motion is found to be free with m, Va, and γ.

Figure 5 illustrates the variations in $R_{DT,c}$, $\lambda_{c}$, and σ as a function of γ for varied estimates of the chemical reacting parameter $K_{RN}$ and the interior heating parameter $S_{N}$. From Figure 5i,ii, we found that with a boost in the values of $K_{RN}$, $S_{N}$, and γ, the critical thermal Rayleigh–Darcy number $R_{DT,c}$ diminishes. This shows that the chemical reacting parameter $K_{RN}$, the interior heating parameter $S_{N}$, and the relaxation parameter γ speed up the beginning of convective activities. This is due to the fact that increasing the chemical reacting parameter $K_{RN}$ and the interior heating parameter $S_{N}$ creates more disturbances by increasing the energy supply to the system. Further, the destabilizing effect of the relaxation parameter γ is found because thermal diffusivity of the system increases with γ (from the definition of γ). From Figure 5iii,iv, it is noted that the critical wave number $\lambda_{c}$ decreases with $K_{RN}$, $S_{N}$, and γ. This illustrates that the magnitude of convection cells increases with $K_{RN}$, $S_{N}$, and γ. From Figure 5v,vi, we detected that the frequency of oscillations σ decreases with accumulating $K_{RN}$ and $S_{N}$, while an opposite result is seen with γ.

Figure 6 exhibits the impact of $R_{DS}$ and Le on the stability of the scheme. From Figure 6i,ii, it is noticed that an improvement in the estimation of $R_{DS}$ and Le is to speed up the marginal and oscillatory patterns of convective motions.

This is on the ground that the disturbance to the arrangement increases with $R_{DS}$ and Le. Furthermore, an increase in the estimate of Le increases the threshold estimate of γ at which the pattern of instability is amended. From Figure 6iii,iv, it is found that the critical wave number $\lambda_{c}$ declines with $R_{DS}$ and Le for a marginal pattern of convection, while a reverse result is seen for an oscillatory pattern of convection. From Figure 6v,vi, it is established that the frequency of oscillations σ shrinks with $R_{DS}$ and Le.

The impacts of the heat capacity ratio m and the Vadasz number Va on $R_{DT,c}$, $\lambda_{c}$, and σ are exposed in Figure 7. From Figure 7i,ii, it is found that $R_{DT,c}$ surges with m for oscillatory pattern of convection, while this result is opposite with Va. This appears that the outcome of m delays the start of an oscillatory pattern of convection. This is due to fact that the energy restoring capacity of arrangement enhances with escalating the heat capacity ratio m.

Further, the Vadasz number Va advances the start of the oscillatory type of movement. A similar result of the Vadasz number Va on the system was also observed by Kumar and Bhadauria [41] and Malashetty and Biradar [76]. From Figure 7iii,iv, it is proven that the critical wave number $\lambda_{c}$ increases with m and Va. The frequency of oscillations σ diminishes with *m*, while this result is opposite with Va as found from Figure 7v,vi. From Figure 7, it is also noticed that increasing Va decreases the threshold estimate of γ at which the pattern of instability alters, while this result is reversed with m.

Figure 8 demonstrates the power of the aspect ratio A on the stability of the system. From this graph, it is found that $\lambda_{c}$ reduces with escalating A. This illustrates that the dimension of convective cells enhances with increasing the aspect ratio A. From Figure 8, it is also found that A has no control on $R_{DT,c}$ and σ.

To observe the effect of $K_{RN},\;S_{N},\;R_{DS},\;Le$, and A on steady-state heat and mass spreads, the Nusselt number Nu and Sherwood number Sh are plotted in Figure 9 as a function $R_{DT}$ for diverse values of these parameters. From this figure, we recognize that if $R_{DT}$ increases from one to five or six times of $R_{DT,c}$, the heat and mass transport increased significantly, and if $R_{DT}$ increases more, it remains moderately constant.

The convective steady mass transfer increased with increasing $K_{RN},\;R_{DS},\;Le$, and A for the slender enclosure (A<1), while it decreased with A for rectangular enclosure A. From Figure 9, it is also found that increases in the values of $S_{N}$, $R_{DS}$, and A for the slender enclosure (A<1) amplify the convective heat transport in the scheme, while for a rectangular enclosure (A>1), convective heat transport decreases with A.

The nonlinear unsteady ordinary differential Equations (47)–(51) are solved numerically by applying the RKF45 method with realistic initial circumstances. The achieved outcomes are presented in Figure 10 and Figure 11. It is noticed that the greatest increase in Nusselt number Nu and Sherwood number Sh appear near to the opening time; it reveals the spatial progress of increasing frequency. Lastly, the oscillations reach the steady situation for a sufficiently large amount of time. From Figure 10 and Figure 11, it is also found that increases in the estimates of $K_{RN},\;S_{N},\;R_{DS},\;R_{DT},\;Le,\;Va,$A for the slender enclosure (A<1) and γ enhance the convective unsteady mass transmission in the arrangement, while it decreases with A for rectangular enclosure (A>1) and m. The convective unsteady heat transportation in the structure increases with increasing $S_{N},\;R_{DS},\;R_{DT},\;Va,$A for the slender enclosure (A<1) and γ, whereas it reduces with A for the rectangular enclosure (A>1) and m.

In Figure 12, a comparison is made between the obtained results and the experimental results offered by Serkitjis [77] for normal fluid. From this figure, it is noticed that the heat spread observed by the experiment are very close to our outcomes if $R_{DT}\geq R_{DT,c}$, whereas for $R_{DT}<R_{DT,c}$, the experimental results are slightly lower, here $R_{DT,c}=4\pi^{2}$.

## 7. Conclusions

The double diffusive convective progress in a Maxwell fluid occupying the interior heat-generating and chemical reacting porous enclosures was investigated analytically as well as numerically taking linear and weak nonlinear stability theories. The considered enclosures were rectangular (A>1), square (A=1), and slender (A<1). The impact of various parameters on the onset of the convective flow, and on the convective heat and mass transports of the system were achieved. The important conclusions are as follows:

- Increasing the chemical reacting parameter $K_{RN}$, the interior heating parameter $S_{N}$, the solute Rayleigh–Darcy number $R_{DS}$, the relaxation parameter γ, the Lewis number Le, and the Vadasz number Va accelerates the onset of double diffusive convective motion, while it delays with increasing the heat capacity ratio *m*.
- The dimension of convective cells enhances by increasing the aspect ratio A, the chemical reacting parameter $K_{RN}$, the interior heating parameter $S_{N}$, and the relaxation parameter γ, while it decreases with the heat capacity ratio m.
- Increasing $R_{DS}$ and Le enhances the size of marginal convective cells, while this result is opposite for oscillatory convection.
- The convective mass transfer in the system is augmented with increasing $K_{RN},\;S_{N},\;R_{DS},\;R_{DT},\;Le,\;Va,$A (for the slender enclosure) and γ, while it reduces with A (for rectangular enclosure) and m.
- The convective heat transport in the system is enhanced with increasing $S_{N},\;R_{DS},\;R_{DT},\;Va,$A (for the slender enclosure) and γ, whereas it reduces with A (for rectangular enclosure) and m.
- The marginal pattern of the convective motion and steady heat and mass transport are observed to be free with m, Va, and γ.

## Figures and Tables

**Table 1:** Comparison of $R_{DT,c}$, $\lambda_{c}$, and σ for different values of γ, $K_{RN}$, and $S_{N}$ at Le=2, m=1.5, A=1, $R_{DS}=3$, $S_{N}=0.5$, $K_{RN}=0.5$, and Va=3.

$K_{RN}$	γ	$S_{N}=0$			$S_{N}=0.5$			$S_{N}=1.0$		
		$R_{DT,c}$	$\lambda_{c}$	σ	$R_{DT,c}$	$\lambda_{c}$	σ	$R_{DT,c}$	$\lambda_{c}$	σ
0	0	33.48	3.14	0.00	32.21	3.11	0.00	30.96	3.07	0.00
	0.3	33.48	3.14	0.00	32.21	3.11	0.00	30.96	3.07	0.00
	0.6	19.48	3.42	1.26	18.85	3.40	1.26	18.23	3.38	1.25
	0.9	10.30	3.35	1.34	9.95	3.33	1.34	9.61	3.30	1.34
0.5	0	32.99	3.13	0.00	31.74	3.09	0.00	30.50	3.06	0.00
	0.3	32.99	3.13	0.00	31.74	3.09	0.00	30.50	3.06	0.00
	0.6	19.10	3.41	1.26	18.49	3.39	1.25	17.88	3.37	1.25
	0.9	9.90	3.33	1.34	9.56	3.31	1.34	9.23	3.29	1.33
1.0	0	32.44	3.11	0.00	31.21	3.08	0.00	29.98	3.04	0.00
	0.3	32.44	3.11	0.00	31.21	3.08	0.00	29.98	3.04	0.00
	0.6	18.70	3.40	1.25	18.09	3.38	1.25	17.49	3.36	1.24
	0.9	9.45	3.30	1.33	9.13	3.28	1.33	8.81	3.26	1.33
1.5	0	31.81	3.09	0.00	30.59	3.05	0.00	29.39	3.02	0.00
	0.3	31.81	3.09	0.00	30.59	3.05	0.00	29.39	3.02	0.00
	0.6	18.24	3.39	1.24	17.65	3.37	1.24	17.07	3.34	1.23
	0.9	8.95	3.27	1.33	8.65	3.26	1.32	8.35	3.24	1.32

**Table 2:** Comparison of $R_{DT,c}$, $\lambda_{c}$, and σ for diverse values of γ, $R_{DS}$, and Le at Le=2, m=1.5, A=1, $R_{DS}=3$, $S_{N}=0.5$, $K_{RN}=0.5$, and Va=3.

$R_{DS}$	γ	Le=2			Le=4			Le=6		
		$R_{DT,c}$	$\lambda_{c}$	σ	$R_{DT,c}$	$\lambda_{c}$	σ	$R_{DT,c}$	$\lambda_{c}$	σ
0	0	37.98	3.10	0.00	37.98	3.10	0.00	37.98	3.10	0.00
	0.3	37.98	3.10	0.00	37.98	3.10	0.00	37.98	3.10	0.00
	0.6	24.37	3.37	1.27	24.37	3.37	1.27	24.37	3.37	1.27
	0.9	15.54	3.29	1.35	15.54	3.29	1.35	15.54	3.29	1.35
2	0	33.82	3.10	0.00	28.95	3.07	0.00	23.18	3.01	0.00
	0.3	33.82	3.10	0.00	28.95	3.07	0.00	23.18	3.01	0.00
	0.6	20.45	3.38	1.26	17.89	3.55	1.14	16.67	3.89	0.96
	0.9	11.56	3.30	1.34	8.43	3.49	1.26	6.20	3.86	1.15
4	0	29.66	3.09	0.00	19.90	3.04	0.00	8.31	2.92	0.00
	0.3	29.66	3.09	0.00	19.90	3.04	0.00	8.31	2.92	0.00
	0.6	16.52	3.40	1.25	11.17	3.71	1.04	7.60	4.22	0.73
	0.9	7.57	3.31	1.33	1.14	3.66	1.20	−4.21	4.21	1.03
6	0	25.50	3.09	0.00	10.85	3.01	0.00	−6.63	2.83	0.00
	0.3	25.50	3.09	0.00	10.85	3.01	0.00	−6.63	2.83	0.00
	0.6	12.60	3.41	1.23	4.27	3.84	0.94	−6.63	2.83	0.00

**Table 3:** Comparison of $R_{DT,c}$, $\lambda_{c}$, and σ for diverse values of γ, m, and Va at Le=2, m=1.5, A=1, $R_{DS}=3$, $S_{N}=0.5$, $K_{RN}=0.5$, and Va=3.

m	γ	Va=2			Va=4			Va=6		
		$R_{DT,c}$	$\lambda_{c}$	σ	$R_{DT,c}$	$\lambda_{c}$	σ	$R_{DT,c}$	$\lambda_{c}$	σ
1	0	31.74	3.09	0.00	31.74	3.09	0.00	31.74	3.09	0.00
	0.3	31.74	3.09	0.00	31.74	3.09	0.00	20.71	3.58	2.28
	0.6	29.01	3.28	0.48	11.93	3.35	1.78	6.29	3.44	2.46
	0.9	16.64	3.23	0.91	5.56	3.30	1.68	1.91	3.38	2.19
1.5	0	31.74	3.09	0.00	31.74	3.09	0.00	31.74	3.09	0.00
	0.3	31.74	3.09	0.00	31.74	3.09	0.00	22.78	3.75	2.07
	0.6	30.36	3.35	0.35	12.56	3.43	1.73	6.66	3.51	2.42
	0.9	17.18	3.28	0.89	5.77	3.34	1.67	1.99	3.41	2.18
2.0	0	31.74	3.09	0.00	31.74	3.09	0.00	31.74	3.09	0.00
	0.3	31.74	3.09	0.00	31.74	3.09	0.00	25.05	3.94	1.87
	0.6	31.74	3.09	0.00	13.26	3.51	1.69	7.08	3.59	2.39
	0.8	21.14	3.35	0.80	7.75	3.42	1.68	3.28	3.47	2.25
	0.9	17.75	3.33	0.86	6.00	3.38	1.65	2.08	3.43	2.17
2.5	0	31.74	3.09	0.00	31.74	3.09	0.00	31.74	3.09	0.00
	0.3	31.74	3.09	0.00	31.74	3.09	0.00	27.48	4.13	1.67
	0.6	31.74	3.09	0.00	14.02	3.60	1.65	7.56	3.67	2.36
	0.8	21.94	3.41	0.76	8.11	3.47	1.66	3.46	3.52	2.23
	0.9	18.37	3.38	0.84	6.26	3.43	1.64	2.19	3.47	2.16

**Table 4:** Comparison of $R_{DT,c}$, $\lambda_{c}$, and σ for diverse values of γ, A, and $R_{DS}$ at Le=2, m=1.5, A=1, $R_{DS}=3$, $S_{N}=0.5$, $K_{RN}=0.5$, and Va=3.

A	γ	$R_{DS}=0$			$R_{DS}=2$			$R_{DS}=4$		
		$R_{DT,c}$	$\lambda_{c}$	σ	$R_{DT,c}$	$\lambda_{c}$	σ	$R_{DT,c}$	$\lambda_{c}$	σ
0.5	0	37.98	6.20	0.00	33.82	6.19	0.00	29.66	6.18	0.00
	0.3	37.98	6.20	0.00	33.82	6.19	0.00	29.66	6.18	0.00
	0.6	24.37	6.74	1.27	20.45	6.77	1.26	16.52	6.79	1.25
	0.9	15.54	6.57	1.35	11.56	6.60	1.34	7.57	6.63	1.33
0.75	0	37.98	4.13	0.00	33.82	4.13	0.00	29.66	4.12	0.00
	0.3	37.98	4.13	0.00	33.82	4.13	0.00	29.66	4.12	0.00
	0.6	24.37	4.49	1.27	20.45	4.51	1.26	16.52	4.53	1.25
	0.9	15.54	4.38	1.35	11.56	4.40	1.34	7.57	4.42	1.33
1.0	0	37.98	3.10	0.00	33.82	3.10	0.00	29.66	3.09	0.00
	0.3	37.98	3.10	0.00	33.82	3.10	0.00	29.66	3.09	0.00
	0.6	24.37	3.37	1.27	20.45	3.38	1.26	16.52	3.40	1.25
	0.9	15.54	3.29	1.35	11.56	3.30	1.34	7.57	3.31	1.33
1.25	0	37.98	2.48	0.00	33.82	2.48	0.00	29.66	2.47	0.00
	0.3	37.98	2.48	0.00	33.82	2.48	0.00	29.66	2.47	0.00
	0.6	24.37	2.70	1.27	20.45	2.71	1.26	16.52	2.72	1.25
	0.9	15.54	2.63	1.35	11.56	2.64	1.34	7.57	2.65	1.33

## Data Availability

Not applicable.

## Nomenclature

A	aspect ratio
c	specific heat $\left(J\cdot {\mathrm{kg}}^{-1}\cdot K^{-1}\right)$
$D_{S}$	solutal diffusivity $\left(m^{2}/s\right)$
g	gravity vector $\left({m/s}^{2}\right)$
$H_{x}$	dimensional Maxwell fluid layer length (m)
$H_{z}$	dimensional Maxwell fluid layer width (m)
K	permeability of the porous matrix $\left(m^{2}\right)$
km	effectual thermal conductivity of the porous matrix $\left({\mathrm{Wm}}^{-1}K^{-1}\right)$
$K_{R}$	chemical reaction rate $\left(s^{-1}\right)$
$K_{RN}$	chemical reacting parameter
Le	Lewis number
m	heat capacity ratio
P	pressure (Pa)
$R_{DT}$	thermal Rayleigh–Darcy number
$R_{DS}$	solute Rayleigh–Darcy number
S	strength of the internal heat supply $\left({\mathrm{Wm}}^{-3}K^{-1}\right)$
$S_{N}$	interior heating parameter
Va	Vadasz number
$V_{D}$	Darcy’s velocity $\left({\mathrm{ms}}^{-1}\right)$
(x,y,z)	space coordinates (m)
**Greek symbols**	
$\alpha_{E}$	effectual thermal diffusivity $\left(m^{2}/s\right)$
$\beta_{\theta }$	the thermal expansion coefficient $\left(K^{-1}\right)$
$\beta_{\phi }$	the solute expansion coefficient
γ	relaxation parameter
$\gamma_{1}$	the stress relaxation $\left(s^{-1}\right)$
λ	dimensionless wave number
μ	viscosity (Pa⋅s)
ρ	density $\left(\mathrm{kg}/m^{3}\right)$
θ	temperature (K)
ϕ	concentration of solute
χ	stream function $\left(m^{2}/s\right)$
ε	the porosity of the porous matrix
σ	enlargement rate of disturbance
τ	time (s)
**Superscripts**	
′	perturbed quantities
∼	dimensionless variables
**Subscripts**	
o	reference estimate
E	effectual estimate
L	estimate at the lower boundary
U	estimate at the upper boundary
b	basic flow
c	critical
